# Supplementary figures and images for: A Study of GUS Expression in Arabidopsis as a Tool for the Evaluation of Gene Evolution, Function and the Role of Expression Derived from Gene Duplication
Source: Plants (Basel). 2023 May 22;12(10):2051. doi: 10.3390/plants12102051 (PMC10221982; doi:10.3390/plants12102051)

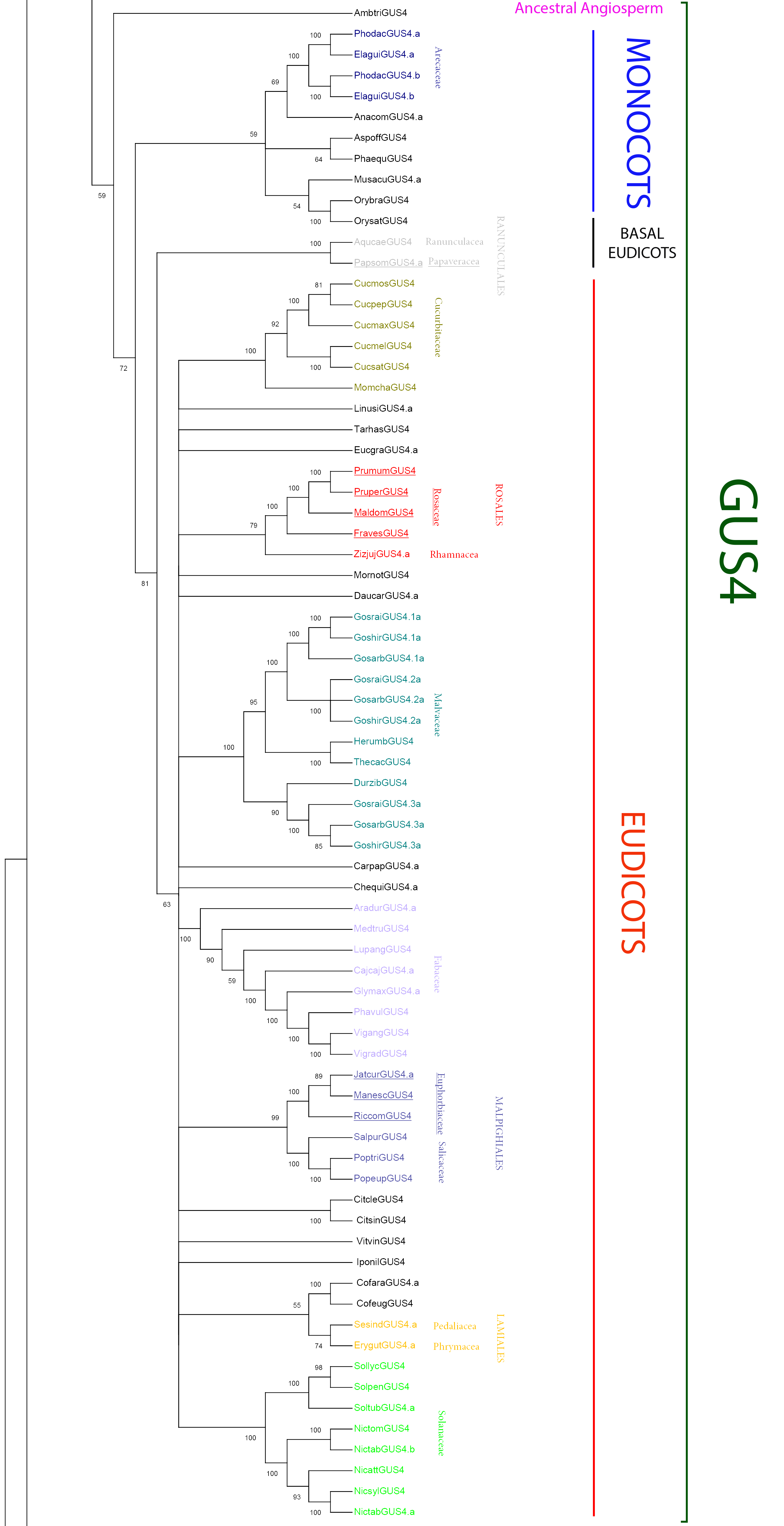

Supplement: Supplementary file 1 [file plants-12-02051-s001.zip › Figure S2.tif]

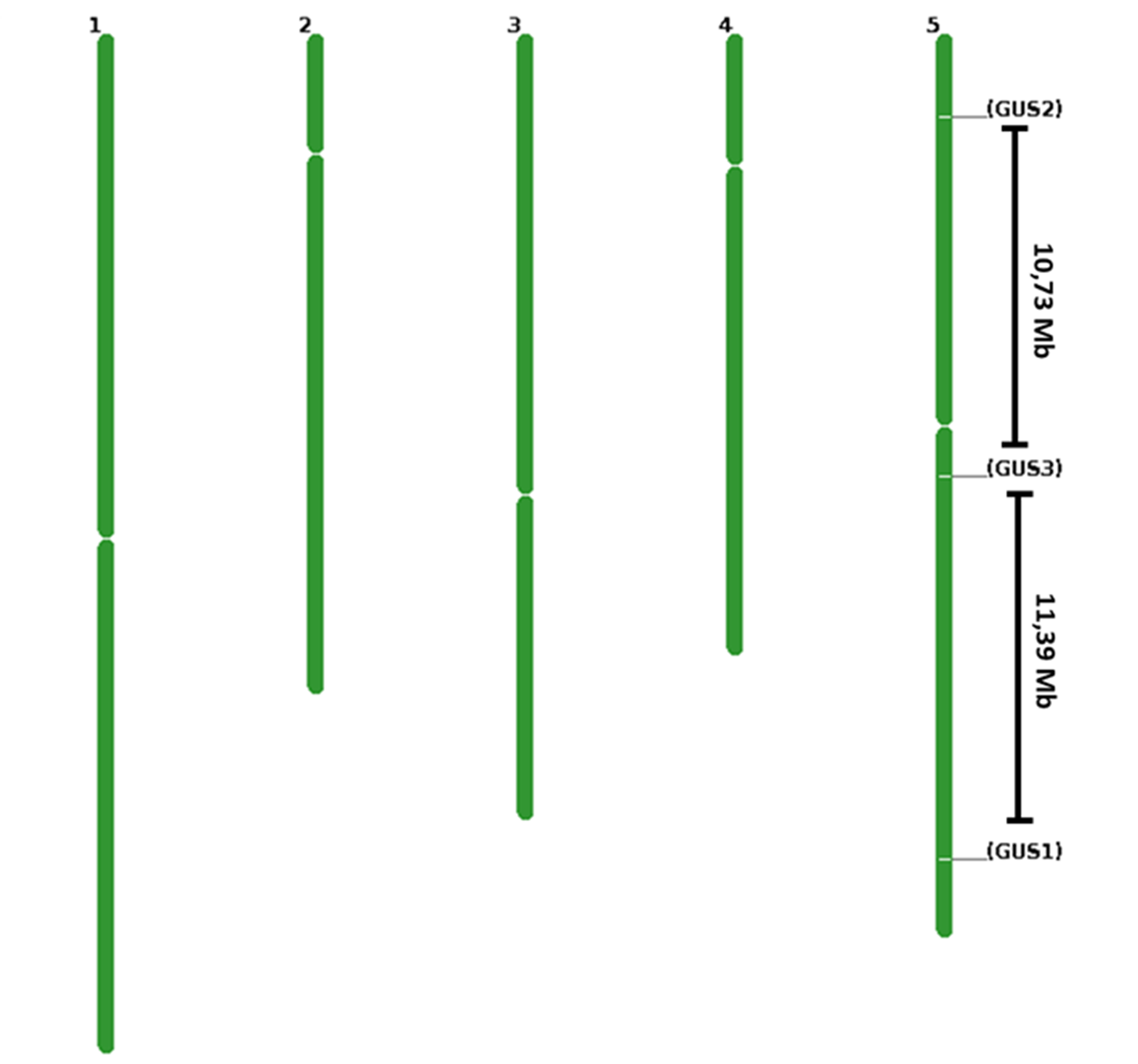

Supplement: Supplementary file 1 [file plants-12-02051-s001.zip › Figure S4.tif]
